# Integrated Short-term Palliative Rehabilitation to improve quality of life and equitable care access in incurable cancer (INSPIRE): a multinational European research project

**DOI:** 10.1177/26323524231179979

**Published:** 2023-06-14

**Authors:** Joanne Bayly, Hilde Hjelmeland Ahmedzai, Maria Grazia Blandini, Barbara Bressi, Augusto Tommaso Caraceni, Joana Carvalho Vasconcelos, Stefania Costi, Stefania Fugazzaro, Monica Guberti, Mai-Britt Guldin, May Hauken, Irene Higginson, Barry J.A. Laird, Julie Ling, Charles Normand, Lise Nottelmann, Line Oldervoll, Cathy Payne, A. Toby Prevost, Guro B. Stene, Elisa Vanzulli, Eduardo Veber, Guillaume Economos, Matthew Maddocks

**Affiliations:** Cicely Saunders Institute of Palliative Care, Policy & Rehabilitation, King’s College London, 5 Bessemer Road, London SE59PJ, UK; Centre for Crisis Psychology, University of Bergen, Bergen, Norway; Fondazione IRCCS Istituto Nazionale dei Tumori, Milano, Italy; Physical Medicine and Rehabilitation Unit, Azienda USL – IRCCS di Reggio Emilia, Reggio Emilia, Italy; Fondazione IRCCS Istituto Nazionale dei Tumori, Milano, Italy; Cicely Saunders Institute of Palliative Care, Policy & Rehabilitation, King’s College London, London, UK; Nightingale-Saunders Clinical Trials and Epidemiology Unit, King’s College London, London, UK; Physical Medicine and Rehabilitation Unit, Azienda USL – IRCCS di Reggio Emilia, Reggio Emilia, Italy; Surgical, Medical and Dental Department of Morphological Sciences, University of Modena and Reggio Emilia, Reggio Emilia, Italy; Physical Medicine and Rehabilitation Unit, Azienda USL – IRCCS di Reggio Emilia, Reggio Emilia, Italy; Research and EBP Unit, Health Professions Department, Azienda USL – IRCCS di Reggio Emilia, Reggio Emilia, Italy; Research Unit for General Practice, Aarhus University, Aarhus, Denmark; Centre for Crisis Psychology, University of Bergen, Bergen, Norway; Cicely Saunders Institute of Palliative Care, Policy & Rehabilitation, King’s College London, London, UK; Western General Hospital and Institute of Genetics and Cancer, University of Edinburgh, Edinburgh, UK; European Association for Palliative Care, Vilvoorde, Belgium; Cicely Saunders Institute of Palliative Care, Policy & Rehabilitation, King’s College London, London, UK; Research Unit for General Practice, Aarhus University, Aarhus, Denmark; The Research Unit, Department of Palliative Medicine, Bispebjerg Hospital, København, Denmark; Centre for Crisis Psychology, University of Bergen, Bergen, Norway; Faculty of Medicine and Health Sciences, Norwegian University of Science and Technology, Trondheim, Norway; European Association for Palliative Care, Vilvoorde, Belgium; Cicely Saunders Institute of Palliative Care, Policy & Rehabilitation, King’s College London, London, UK; Nightingale-Saunders Clinical Trials and Epidemiology Unit, King’s College London, London, UK; Centre for Crisis Psychology, University of Bergen, Bergen, Norway; Department of Neuromedicine and Movement Science, Norwegian University of Science and Technology, Trondheim, Norway; Fondazione IRCCS Istituto Nazionale dei Tumori, Milano, Italy; European Cancer Patient Coalition, Brussels, Belgium; Centre Hospitalier Lyon-Sud, Palliative Care Centre, Pierre-Benite, France; Cicely Saunders Institute of Palliative Care, Policy & Rehabilitation, King’s College London, London, UK

**Keywords:** incurable cancer, palliative care, palliative rehabilitation, quality of life, rehabilitation

## Abstract

**Background:**

Disability related to incurable cancer affects over a million Europeans each year and people with cancer rank loss of function among the most common unmet supportive care needs.

**Objectives:**

To test the clinical and cost-effectiveness of an integrated short-term palliative rehabilitation intervention, to optimise function and quality of life in people affected by incurable cancer.

**Design:**

This is a multinational, parallel group, randomised, controlled, assessor blind, superiority trial.

**Methods:**

The INSPIRE consortium brings together leaders in palliative care, oncology and rehabilitation from partner organisations across Europe, with complementary expertise in health service research, trials of complex interventions, mixed-method evaluations, statistics and economics. Partnership with leading European civil society organisations ensures citizen engagement and dissemination at the highest level. We will conduct a multinational randomised controlled trial across five European countries, recruiting participants to assess the effectiveness of palliative rehabilitation for people with incurable cancer on the primary outcome – quality of life – and secondary outcomes including disability, symptom burden and goal attainment. To support trial conduct and enhance analysis of trial data, we will also conduct: comparative analysis of current integration of rehabilitation across oncology and palliative care services; mixed-method evaluations of equity and inclusivity, processes and implementation for the intervention, at patient, health service and health system levels. Finally, we will conduct an evidence synthesis, incorporating INSPIRE findings, and a Delphi consensus to develop an international framework for palliative rehabilitation practice and policy, incorporating indicators, core interventions, outcomes and integration methods.

**Scientific contribution:**

If positive, the trial could produce a scalable and equitable intervention to improve function and quality of life in people with incurable cancer and reduce the burden of care for their families. It could also upskill the practitioners involved and motivate future research questions. The intervention could be adapted and integrated into different health systems using existing staff and services, with little or no additional cost.

## Background – what are novel important questions the project seeks to answer? What is the current state of science?

Cancer is one of the main causes of illness, burden and death in Europe. The Joint Research Centre (JRC) of the European Union (EU) estimated 2.7 million new cancer cases and 1.3 million deaths in 2020 in people over 65 years of age.^1^ For all cancers, between 53–79% of men and 41–62% of women are diagnosed with incurable disease,^2^ and treatment may be life-prolonging but not curative. Survival rates are increasing overall, but least so for older people and those with multimorbidity, which are both growing populations.^3^ The total cost of cancer in Europe reached €199 billion in 2018, with approximately equal costs within and outside health care systems.^4^

Disability related to incurable cancer affects over 1 million Europeans each year, and recent global estimates suggest a loss of 382 disability-adjusted life years per 1000 individuals.^5,6^ People with cancer rank loss of function among the most common unmet supportive care needs.^7–10^ This can occur because of the disease, its treatment and related symptoms (e.g. breathlessness, pain, fatigue)^11^ and syndromes (e.g. cachexia, sarcopenia).^12^ Over time, loss of function, for example, impaired mobility or worsening symptoms on movement, results in people losing independence in valued roles and routines. They experience increasing difficulty in managing usual household and social activities and self-care. One-third of adults with cancer require assistance to perform basic activities like washing and dressing, and half need help with extended activities like shopping and transportation.^9^ Disability reduces quality of life and well-being.^7–10^ It increases the care provision required from informal carers, including family members^13^ and formal care services including demand for hospital or nursing care.^4^ Disability related to daily activity is closely related to unplanned hospital admissions and mortality.^12^

Rehabilitation in palliative care aligns with modern definitions of rehabilitation. It involves processes and interacting interventions delivered in health and social care systems to optimise functioning and reduce disability in individuals to support them to achieve and maintain optimal functioning in their physical and social environment.^14^ It can empower people with incurable cancer to actively manage their condition themselves, enabling them to live fully and enjoy the best quality of life possible.^15,16^ It aims to reduce symptoms and help people to stay independent and socially active,^17,18^ including towards the end of life.^19^ The World Health Organization (WHO) policy on Universal Health Coverage (UHC) states that both rehabilitation and palliative care are essential quality health services^20^ and recommends both are integrated within and between primary, secondary and tertiary health systems using a multiprofessional workforce. While rehabilitation services are well integrated into routine care for people with chronic respiratory,^21^ cardiac^22^ and stroke conditions,^23,24^ this is not currently the case for people living with incurable cancer. Despite the strong evidence for need and increased access to palliative care services, access to routinely provided integrated rehabilitation for people with incurable cancer remains limited and varied across and within European countries in both goals, delivery methods and organisational structures in primary and secondary health care. The reasons are multifold, including the historical development of rehabilitation and palliative care as specialities and the organisation and provision of oncology services.^25^ New, innovative models of accessible and equitable rehabilitation are required to meet the unique needs of people living with incurable cancer.

Exercise-based rehabilitation interventions for patients with cancer, underpinned by exercise guidance, are becoming the established standard of care to maintain physical function and prevent and treat health-related outcomes including symptoms and to preserve function in activities of daily living.^26^ Exercise training is generally safe for people with cancer. Supervision from experts with a prescription that combines aerobic and resistance training is recommended. Most exercise evidence, however, arises from people with early-stage cancer, often following adjuvant treatment, and far less relates to people with incurable cancer.^27–29^ Low adherence^30^ and high attrition are common in exercise studies for people with incurable disease.^31^ The potential gains may be difficult to realise for people with incurable cancer or restricted to physical fitness.^32^ Supervised, intensive programmes (e.g. two to three sessions each week) using specialist equipment are not always acceptable or accessible to people living with life-limiting illness. Many people with incurable cancer and their clinicians perceive rehabilitation to be burdensome and not aligned with their priorities, of no benefit and too difficult to access during busy treatment schedules.^33–35^

Three recent trials in people with incurable cancer have tested different models of predominantly exercise-based rehabilitation interventions. Positive trials include a three-arm tele-rehabilitation intervention in which a remotely supervised exercise intervention with automated symptom monitoring with (arm 3) and without (arm 2) nurse led remote pain management *versus* an automated monitoring control group (arm 1) (N516) achieved a larger than minimally important difference improvement in the Activity Measure for Post-Acute Care, Computer Adaptive Test, Basic Mobility Bank (Standard Mean Difference (SMD) of arm 2 versus arm 1 was 1.3; 95% confidence interval (CI) = [0.08 to 2.35] with no significant difference between arm 1 and arm 3. EQ-5D-3L quality of life score only improved significantly for the tele-rehabilitation arm 2; 0.04 [0.004 to 0.071]).^36^

A centre-based multiprofessional, multimodal palliative rehabilitation programme integrated in an oncology clinic plus standard care *versus* standard care alone (N288) favoured the intervention arm with a between-group difference of 3.0, 95% CI = [0.0 to 6.0]; effect size is 0.3 for the EORTC QLQ C-30.^37^ A supervised, exercise-based intervention with nurse-led symptom control delivered by telephone *versus* standard care (N92) did not achieve improvements in the primary outcome at 9 weeks (6-min walking distance, m) SMD −25.4, 95% CI = [−64.0 to 13.3] but at 6 months found improvements in both quality of life (mean difference in FACT-L Trial Outcome Index 10.4, 95% CI = [4.0 to 16.9]) and in symptoms as measured by the MDASI-LC −2.23, 95% CI = [−3.56 to −0.90].^38^

A fourth trial, testing a tailored occupational therapy intervention compared with usual care at 12 weeks (N242), did not demonstrate improvements in motor or process skills in activities of daily living (in both motor ability and process ability domains).^39^ The single country settings and limited evaluation of health economics (confined to the US setting) limit the generalisability, scalability and sustainability of the interventions of these trials.^40^ Practice changing evidence for person-centred, goal-orientated rehabilitation interventions is still required.

To address the gaps in evidence and to make palliative rehabilitation part of routine care for people with incurable cancer, palliative care and palliative rehabilitation researchers and clinicians from seven EU countries (England, Scotland, France, Norway, Denmark, Italy and Belgium) formed a partnership to refine and test an innovative, scalable model of rehabilitation for people with incurable cancer. The ‘Integrated Short-term Palliative Rehabilitation to improve quality of life and equitable care access in incurable cancer’ (INSPIRE) project aims to address the specific functional needs and goals of people living with incurable cancer and to be implementable in oncology, palliative and primary care settings. It builds on considerable experience and preliminary data following the UK Medical Research Council guidance for complex interventions.^41^ This includes a systematic review,^42^ exploring the application of behaviour change approaches in empirical rehabilitation studies and focus groups with patients, family members and clinicians,^43^ where participants prioritised prompt, short-term input and involvement of carers.

To optimise access, inclusivity and equity, the INSPIRE model of rehabilitation is individualised and tailored to each person’s concerns, priorities and goals. Rehabilitation components are selected by a rehabilitation practitioner (RP) and the person to address the natural progression of their disease, and how they are living with their illness, to achieve outcomes that are meaningful to them in their unique context. It combines previously tested symptom self-management, physical activity and exercise and goal-orientated approaches.^43,44^ It has been designed to be delivered across settings as a parallel approach to oncology and palliative care.^45^ The project is timely as the science supporting rehabilitation as helpful towards end of life is growing but definitive evidence is lacking.

## Aim and objectives of the project and its studies

### Aim

We aim to identify an effective model of rehabilitation that can be delivered as part of routine care for people with incurable cancer, which integrates with oncology and palliative care.

### Objectives

**Figure 1.**
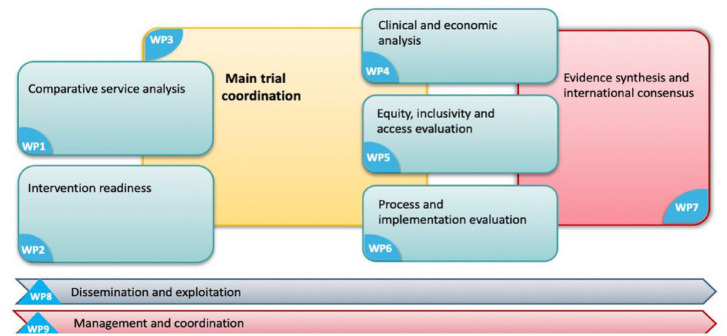
Work packages.

The project protocol consists of eight objectives delivered across work packages (WPs) as shown in Table 1. A ninth work package will deliver overall project and scientific management and coordination (Figure 1).

**Table 1.** Specific objectives.

Objective number	Objective	Work package
1	To compare models and levels of integration between rehabilitation, oncology and palliative care services across different health care systems in Europe	Comparative health service analysis
2	To develop implementable materials and training for a palliative rehabilitation intervention for adults with incurable cancer and functional limitation across five European countries	Intervention readiness
3	To set up and conduct a randomised single-blind multicentre trial to assess the clinical effectiveness of palliative rehabilitation over 8 weeks on quality of life, disability, symptom burden and goal attainment for patients with incurable cancer	Main trial coordination
4	To assess the cost-effectiveness of palliative rehabilitation from a health care and societal perspective, focusing on hospital treatment and care costs, ambulatory care costs and costs to informal caregivers, presenting cost-utility estimates as well as cost-effectiveness in terms of changes in primary outcome	Clinical and economic analysis
5	To determine equity, access and patient experience of the intervention, across different cultures, socio-economic and other groups, considering gender, age, religious, cultural and personal belief	Equity, inclusivity and access evaluation
6	To evaluate whether the palliative rehabilitation intervention was successfully implemented, identifying factors contributing to successful integration with existing services, fidelity of intervention delivery and clinical effectiveness	Process and implementation evaluation
7	To develop a framework for clinical practice and policy in palliative rehabilitation for people with incurable cancer, incorporating referral triggers, core working practices and outcomes, and integration with oncology and palliative care services	Evidence synthesis and international consensus
8	To disseminate findings of the INSPIRE project to patients, clinicians, health and social care professionals, care providers, policymakers, commissioners, governments and the public to change current practice, with impact beyond the life of the grant	Dissemination and exploitation

## Methods – what are the study design and methods, the cross-national consortium membership, public and patient involvement?

### Design

Mixed-method study with a sequential exploratory design whereby supporting studies are included to explore and describe the results of the multinational randomised controlled trial – and whereby each WP has its own design.

### The INSPIRE consortium

The INSPIRE consortium brings together leaders in palliative care, oncology and rehabilitation from Nordic, Southern and Northern European countries with complementary expertise in applied and health services research, trials of complex interventions, mixed-method evaluations, statistics and health economics. Consortium members lead work packages to achieve the objectives described in Table 1, beginning in September 2022 and completing in September 2026. With project management and scientific coordination shared across French and English partners, each work package supports the design, conduct and evaluation of, and dissemination of findings from, a multinational randomised controlled trial (WP3, WP4).

The consortium partners include the European Cancer Patient Coalition (ECPC) as the largest European association for people with cancer and the European Association of Palliative Care, and in the United Kingdom, the Cicely Saunders Institute Public Involvement Forum, which has members from across the United Kingdom. Citizen, civil society, professional and end-user engagement has already informed the design of the INSPIRE project, and the project was discussed at two public and patient co-design workshops. Active involvement of these stakeholders is planned in each of the study work packages, to ensure acceptable and appropriate research practices, collaborative working and transparency about project processes and decisions. Citizens will be represented on the trial steering committee and at Consortium General Assembly and Scientific Meetings. Between the ECPC and partner sites, we have an excellent platform and clear ambition to expand, diversify and increase the opportunities for and impact of citizen engagement across all aspects of the INSPIRE project.

#### Project hypothesis

Our hypothesis is that our model of palliative rehabilitation, delivered over 8 weeks in addition to usual care, will lead to clinically meaningful improvements in the primary outcome, health-related quality of life as measured by FACT-G,^46^ and secondary outcomes: disability, symptom burden and goal attainment. This may lead to a reduction in costs of hospital and ambulatory care, costs to informal caregivers, cost-utility measures and costs related to changes in the primary outcome. Primary and secondary outcomes will be measured using validated tools with demonstrated ability to capture change in this population.

#### Trial protocol

After ethical approval is obtained, the trial protocol, following SPIRIT guidelines,^47^ will be publicly registered and published. It will include all research processes relating to the main trial and analysis (WP3, WP4), the equity, access and inclusion evaluation (WP5) and the process and implementation evaluation (WP6). Ethical approval will first be obtained from the Sponsor site. Once obtained, each partner involved in data collection will obtain ethics approval from their local ethical committee before the start of the trial. The results of the ethical committees’ judgements will be collected by the WP3 lead for reporting to the Research Executive Agency. Informed consent to participate in the trial will be obtained from all participants before enrolment on the trial.

To ensure protocol fidelity is maintained, an Intervention Manual and training sessions will be provided for trial teams and RPs delivering the intervention at each participating site. The manual and training will be provided and delivered by the WP2 and WP3 teams before and during the trial. We aim to recruit from oncology and palliative care services and tumour groups in which existing rehabilitation services are limited. This will ensure high fidelity as intervention participants have access to finite rehabilitation resources. In addition, partner leads and project managers from each site will sit on the Trial Management Group and will receive reports from independent Trial Steering and Data Management Committees.

#### The INSPIRE intervention

In brief, the INSPIRE intervention comprises up to three manualised sessions (face to face and by telephone) delivered by an expert RP (physiotherapist, occupational therapist, dietitian, speech and language therapist or rehabilitation nurse), delivered in addition to standard oncology and specialist palliative care. Core components of the intervention will focus on supporting people to self-manage function limiting symptoms^37,38,48–50^ as well as optimising physical activity levels and fitness^31,51–55^ and participation in family and social life.^56–59^ We will use explicit behaviour change techniques with goal setting and action planning. The RP will work in partnership with the person with incurable cancer, and their family/caregivers, to support and optimise their independence and interdependence. Sessions will focus on outcomes identified by each person as important to them and the intervention allows for individual tailoring and flexibility in timing and content.

#### Supporting studies

Within the overall framework, supporting studies will be conducted to optimise the design of the trial and to increase the interpretation, learning and impact of the trial results.

A comparative analysis of health services across the six countries recruiting to the trial (England, Scotland, France, Norway, Denmark and Italy) will address the sparse knowledge that exists on how rehabilitation is integrated into cancer and palliative care services across different health care systems in Europe (WP1). This includes a document analysis of national standards and guidelines, supported by an online survey and stakeholder interviews to understand variation in practice, referral criteria and patient pathways, and offer insight into potential integrations between general oncology, rehabilitation and palliative care services. This work will also identify opportunities to enhance practices and optimise recruitment processes and interpretation of the results, including defining key cost drivers within each country.

To develop readiness to deliver the intervention, WP2 will refine and prepare the INSPIRE Intervention Manual and training sessions for RPs. Culturally congruent participant facing resources and materials will be compiled in the languages of each site.

A mixed-methods approach, collecting and analysing quantitative and qualitative interview data, will be used to conduct an evaluation of equity, inclusivity and access (WP5) and a robust implementation process evaluation (WP6) alongside the trial. We plan to investigate how social inequality affects access and outcomes across and within trial site countries, to understand how social and psychological factors may affect trial enrolment and outcomes by exploring how age, gender, cultural background, socioeconomic position, cultural or personal beliefs or comorbidities play a role in patients accepting the invitation to participate and trial outcomes. For example, there are several gender disparities in prevalence and impact of cancer on patients and their families. Cancer affects men slightly more than women, with 54% of new cases and 56% of deaths,^60^ while disability-adjusted life years lost per cancer case are slightly larger for women.^61^ Women also disproportionally take on informal caring responsibilities: around two-thirds of all informal carers are women.^62^ Considering gendered experiences and outcomes within the INSPIRE project is therefore highly relevant. In response to potential for inequity in intervention benefit relating to sex and gender and to understand gender differences in access and patient experiences, we have specifically incorporated analyses of these factors within Objective 5 (WP5) and Objective 6 (WP6). For quantitative components, sex and gender variables will be included as relevant to explore differences in outcomes and access. For qualitative components, gender will be considered within data collection and analysis: purposive sampling will be used to ensure representation of participants with different genders and reflexive practice considering the influence of the researchers’ personal characteristics on interpretations (including gender) will be undertaken. This mixed-methods approach will support identification of potential mechanisms, mediators and moderators of access and treatment effect relating to person, intervention and service characteristics, and cross-country and within-country barriers and facilitators to access and equitable delivery of the intervention. Not all patients report good experience from rehabilitation and palliative care and access and equity challenges are apparent, for example, according to diagnostic groups and tumour types. Inequality in health care applies especially to vulnerable groups of patients with multimorbidity, socioeconomic disparities or cultural and personal disadvantages.^63,64^

Our implementation process evaluation will utilise implementation science methodologies^65–67^ to evaluate if the findings can be confidently attributed to the intervention as delivered.^68^ The evaluation will assess uptake, reach and fidelity of intervention delivery and explore how the trial processes and intervention components were received and experienced by patients, family caregivers and health care professionals. Analysis of quantitative and qualitative data will identify potential mechanisms of action, and mediators and moderators of effect relating to person, intervention, service and contextual characteristics. The evaluation will identify the implementation elements needed to facilitate future implementation of the intervention in real-world settings with maximal impact.^69^ If it is found to be effective, we will develop intervention implementation guidance and training resources based on the findings of the main trial, the findings of the equity, inclusivity and access study and the process implementation evaluation, to support equitable delivery and scaling up of the intervention across varied health care contexts.

Finally, we will also conduct an evidence synthesis and international consensus setting exercise to incorporate the findings of INSPIRE with wider research in an International Framework for Palliative Rehabilitation (WP7). Planned and purposeful dissemination (WP8) will ensure that guidance and training resources and the International Framework for Palliative Rehabilitation are made widely available to support better provision of functional-orientated care for people with incurable cancer across Europe and beyond.

## Scientific contribution

Our ambition is to produce an innovative model of person-centred palliative rehabilitation that can be integrated into routine care for people with incurable cancer. To achieve this, INSPIRE convenes a unique consortium of experts in palliative rehabilitation, palliative care, rehabilitation clinicians, clinical trials researchers, mixed-method researchers and health economists from across Europe. If successful, the trial could demonstrate how palliative rehabilitation can be integrated into cancer care to improve the quality of life for people with incurable cancer and their informal carers. It may provide an exemplar applicable to other incurable diseases. The integrated short-term palliative rehabilitation implementation guideline, based on the project findings, process evaluation and stakeholder engagement, will describe in detail the processes and resources needed to achieve the expected outcomes for patients across different health services and systems.

Irrespective of the trial findings, the project will have significant value to the field. The collaboration will increase the profile of rehabilitation and create a network to act as a base for further research to improve care for people with incurable cancer. The comparative analysis of health services will highlight differences and variation in practice on how palliative rehabilitation services are organised, to assist their integration into health systems. The International Consensus will develop and agree an internationally applicable framework for palliative rehabilitation for incurable cancer. The INSPIRE project has potential to reduce health-related suffering and improve well-being and quality of life for cancer patients in need of supportive, palliative or end of life care as well as for their family caregivers.

The primary objective of the intervention is to improve quality of life and functional well-being for people with incurable cancer and to reduce the disease burden. People with incurable cancer want to live well and maintain normality for as long as possible, retaining their independence in functional roles and activities, and avoiding feeling like a burden on their families and community.^57,70^ If effective, the INSPIRE palliative rehabilitation intervention will support people to stay well, with optimal function, by mitigating the impact of symptoms, psychological distress and physical deconditioning associated with incurable cancer and oncology treatments. It brings together the values and aims of both palliative care and rehabilitation to delay or mitigate the onset of loss of function and disability through self-management of symptoms, physical activity levels and fitness and social participation. These components have a direct effect on people’s capacity, opportunity and motivation^71^ to manage daily activities in the home and community, both independently and interdependently with support from family caregivers. The intervention is underpinned by robust and theoretically informed development^42,43^ and feasibility work^44^ following established guidelines.^72^ As well as supporting people to restore and maintain function, it will help them to prepare for, adapt to and compensate for, losses in function.^15^ INSPIRE addresses barriers to performance of functional activities and social participation during periods of physical deterioration and decline.^16^ It will advance the reach of rehabilitation across the course of a person’s illness, when they no longer have the life expectancy or physical capacity to benefit from exercise-based interventions offered during earlier stages of illness. This is a major advance in current usual practice.

If effective and cost-effective, INSPIRE will provide a model of more accessible and equitable rehabilitation for people with incurable cancer with evaluated intervention materials, processes and educational resources to support implementation. The findings from the development work and feasibility testing tell us that people with incurable cancer prioritise flexible, accessible and tailored models of rehabilitation that minimise the frequency of appointments at health care settings.^43^ The INSPIRE intervention is designed to facilitate earlier and better access to targeted rehabilitation components that address immediate functional needs without committing people to prolonged interventions or to travelling to conventional rehabilitation centres. It achieves this through integration with cancer and palliative care services in community settings, general hospitals and specialist cancer services, so people can access rehabilitation as soon as they are diagnosed with incurable disease. The intervention is short-term and flexible and can be delivered face to face in health care clinics that people are already attending, their homes, *via* telephone or remote video link. Models of exercise-based cancer rehabilitation, while suitable for people on curative treatment pathways, do not address the range of functional needs experienced by people with incurable cancer.^26,35,73,74^ Integration with existing and available health and nonhealth care services in the local community facilitates the provision of tailored ongoing support after the intervention through signposting and onward referrals. These integration practices may achieve beneficial outcomes for patients and health services at each site after the completion of this project.

INSPIRE is designed to be implemented by a range of rehabilitation professionals (including from physiotherapy, occupational therapy, dietetics, speech and language therapy or nursing) with additional training in palliative rehabilitation. As the rehabilitation workforce capacity varies across countries, and where RPs may come from differing professional groups and be located across cancer, palliative care or primary care settings, the flexible components and processes entailed in the intervention have greater potential to be implemented across international health systems. As a short-term intervention, it has greater potential, if effective, to be scalable and sustainable. A key component of the intervention is to refer or signpost patients and family caregivers to appropriate and available health, social and community services already existing in their locality to provide ongoing support addressing their needs, priorities and goals. Optimising patients’ access to these services makes it more feasible to sustain beneficial outcomes following delivery of the intervention.

The intervention could be low cost or cost neutral as it is implemented through training existing staff with materials that are readily available and manuals. If the intervention is found effective, the manuals will support training of existing staff beyond the trial sites. An economic analysis will determine the economic benefits of the intervention and whether it decreases in-patient hospital stays^36^ and wider societal costs through assessment of caregiving requirements in the community and home setting. Policymakers will have access to information on the clinical and cost-effectiveness of palliative rehabilitation described in their terms, for example, QALYs, and best practice examples. Access to cost-effective, function-orientated care has the potential to reduce the burden and financial impact of caregiving for family caregivers and other health and social care costs associated with informal care.^4,58,75^ This has not been reported in previous trials of rehabilitation in this population,^36–39^ although it is well established that family caregivers experience considerable burden when providing informal care for someone with incurable cancer. This relates to the direct care and support they provide for their family member,^13^ interference in their lifestyle,^76^ and the impact on their own health needs and disability.^77^

The findings from this project will have an impact on commissioners, clinical services and policymakers beyond the lifetime of the project. If effective, it will provide health systems with evidence to design improved, potentially cost-effective services through the integration of palliative rehabilitation into their own oncology and palliative care services.

The comparative service analysis will enhance information about, and access to, rehabilitation for people with incurable cancer, regardless of the results of the trial. Through our stakeholder analysis, a directory of cancer rehabilitation services will be produced, including generalist and nonhealth community rehabilitation providers accessed by people with incurable cancer in the consortium countries. None of the WHO atlas of palliative care, the Centeno (Spain) mapping of palliative care availability or the European Parliament report on Palliative care in the European Union include rehabilitation^78^ and so this will be a significant addition to the field.

Based on the trial findings, we will develop INSPIRE intervention implementation guidelines and evaluation and training resources, localised for each country in the trial, to support equitable delivery of the intervention across varied health care contexts. This will facilitate escalation of the expected beneficial study outcomes for patients and health services. Our outputs, including an evaluated intervention manual and training resources for practitioners, will give health care providers the tools to use the intervention and will be a contribution to integrated working across service boundaries beyond the life of the trial. The production of an international consensus framework will set out detailed policy and practice recommendations relating to referral triggers, integrated working, core interventions and outcomes. Dissemination work with stakeholders including practitioners, commissioners and funders will contribute to escalation of the project outcomes.

INSPIRE will also have a considerable scientific impact. It will contribute to a much-needed body of evidence relating to rehabilitation for people with incurable cancer. The project will inform future research in this area by producing quantitative and qualitative academic reports including proposed and evaluated intervention mechanisms, mediators of action and moderators of effect. The academic and lay reports will be published in open-access journals and on the project website and will summarise contextual and cultural data at each site, adding knowledge on cross-cutting barriers and enablers for equity, inclusivity and access that could be translatable to other services for people with other incurable health conditions.

The outcomes of the INSPIRE study will ideally have a wider public and societal impact. The project will support social understanding about living well with incurable disease. It will help dispel the belief that there is an inevitable and continuous decline in function for people with incurable cancer. It will increase our understanding into the relationship between functioning and quality of life. INSPIRE will work with the ECPC so that members of the public and patients are involved at a high level in the study work packages. This will establish pathways to impact to ensure that the results of the project are available to cancer patients and their families throughout Europe. The project outputs and resources will facilitate provision of more choice for patients. Increased demand for palliative rehabilitation from patients and their families and organisations representing their needs will help speed up its integration into cancer care. Together with the European Association for Palliative Care (EAPC)-instituted Rehabilitation Taskforce, they will be a powerful tool for advocacy to policymakers.

## Conclusion

This multinational European research project builds on the considerable experience of the Project Consortium members and preliminary data. It is timely because despite widespread belief that rehabilitation is helpful towards the end of life, definitive evidence is lacking. The intervention responds to needs identified as important by people living with incurable cancer and focuses on each person’s concerns, priorities and goals, which improves access and equity. If positive, the trial could result in a scalable and equitable intervention that improves function and quality of life in people with incurable cancer, and which reduces the burden of care for their families. The intervention will be adaptable for integration into different health systems using existing staff and services. If the INSPIRE model of rehabilitation is found to be as clinically effective as models of rehabilitation designed for people living with chronic long-term conditions, it is likely to be as cost-effective and sustainable.

## Supplemental Material

sj-doc-1-pcr-10.1177_26323524231179979 – Supplemental material for Integrated Short-term Palliative Rehabilitation to improve quality of life and equitable care access in incurable cancer (INSPIRE): a multinational European research project Click here for additional data file. Supplemental material, sj-doc-1-pcr-10.1177_26323524231179979 for Integrated Short-term Palliative Rehabilitation to improve quality of life and equitable care access in incurable cancer (INSPIRE): a multinational European research project by Joanne Bayly, Hilde Hjelmeland Ahmedzai, Maria Grazia Blandini, Barbara Bressi, Augusto Tommaso Caraceni, Joana Carvalho Vasconcelos, Stefania Costi, Stefania Fugazzaro, Monica Guberti, Mai-Britt Guldin, May Hauken, Irene Higginson, Barry J.A. Laird, Julie Ling, Charles Normand, Lise Nottelmann, Line Oldervoll, Cathy Payne, A. Toby Prevost, Guro B. Stene, Elisa Vanzulli, Eduardo Veber, Guillaume Economos and Matthew Maddocks in Palliative Care and Social Practice

## Declarations

**Ethics approval and consent to participate:** Ethical approval was not required or sought for this work.

**Consent for publication:** All authors have provided their consent for publication.

**Availability of data and materials:** Further information about the project can be obtained from the project website INSPIRE Home – INSPIRE (palliativeprojects.eu).
